# Rolled-up tubes and cantilevers by releasing SrRuO_3_-Pr_0.7_Ca_0.3_MnO_3 _nanomembranes

**DOI:** 10.1186/1556-276X-6-621

**Published:** 2011-12-07

**Authors:** Christoph Deneke, Elisabeth Wild, Ksenia Boldyreva, Stefan Baunack, Peter Cendula, Ingolf Mönch, Markus Simon, Angelo Malachias, Kathrin Dörr, Oliver G Schmidt

**Affiliations:** 1 Laboratorio Nacional de Nanotecnologia, Rua Giuseppe Máximo Scolfaro 10000, Campinas, São Paulo, 13083-100, Brazil; 2 Institute for Integrative Nanosciences, IFW Dresden, Helmholtzstrasse 20, Dresden, 01069, Germany; 3 Institute for Metallic Materials, IFW Dresden, Helmholzstrasse 20, Dresden, 01069, Germany; 4 Institute of Microstructure Technology (IMT), Karlsruhe Institute of Technology (KIT), Hermann-von-Helmholtz-Platz 1, Eggenstein-Leopoldshafen, 76344, Germany; 5 Departamento de Física, Universidade Federal de Minas Gerais, CP 702, Belo Horizonte, Minas Gerais, 30123-970, Brazil; 6 Institute for Physics, Martin Luther University (MLU) Halle-Wittenberg, Von-Danckelmann-Platz 3, Halle, 06120, Germany

**Keywords:** rolled-up nanotubes and microtubes, freestanding membranes, ferroic oxides, strain engineering

## Abstract

Three-dimensional micro-objects are fabricated by the controlled release of inherently strained SrRuO_3_/Pr_0.7_Ca_0.3_MnO_3_/SrRuO_3 _nanometer-sized trilayers from SrTiO_3_(001) substrates. Freestanding cantilevers and rolled-up microtubes with a diameter of 6 to 8 μm are demonstrated. The etching behavior of the SrRuO_3 _film is investigated, and a selectivity of 1:9,100 with respect to the SrTiO_3 _substrate is found. The initial and final strain states of the rolled-up oxide layers are studied by X-ray diffraction on an ensemble of tubes. Relaxation of the sandwiched Pr_0.7_Ca_0.3_MnO_3 _layer towards its bulk lattice parameter is observed as the major driving force for the roll-up of the trilayers. Finally, μ-diffraction experiments reveal that a single object can represent the ensemble proving a good homogeneity of the rolled-up tubes.

**PACS: **81.07.-b; 68.60.-p; 68.37.Lp; 81.16.Dn.

## Background

Perovskite oxides have become a fascinating class of materials because of the wide variety of electronic properties including an intriguing ferroic (magnetic or ferroelectric) response for potential use in memory or sensor applications. At the same time, an epitaxial strain has been demonstrated to massively change the fundamental properties of such oxides, in particular, affecting their electronic behavior [1-4]. A recent sensor design includes freestanding cantilevers for electromechanical devices [3]. An elegant way to form three-dimensional structures based on the release and deterministic rearrangement of two-dimensional films has been established over the last years [5-7]. An inherently strained layer stack is deposited on top of a sacrificial layer (or substrate) and is released by selective removal of this sacrificial layer. Due to cunning strain design and patterning, the layer stack bends up forming cantilevers or rolls up into nano- and microtubes. The technique has been employed to form fluidic systems [8], optical resonators [9-11], microtube lasers [12], metamaterial waveguides [13], and even microrobots [14,15] from various material systems [16,17]. Due to the strain relaxation driving the bending and roll-up processes, the three-dimensional micro-objects exhibit a unique strain state [18], influencing the properties of the microtubes [19].

In this work, an approach for the fabrication of three-dimensional micro-objects (freestanding cantilevers, rolled-up microtubes) from perovskite oxides, i.e., ferromagnetic SrRuO_3 _[SRO] known for its chemical stability [20] and antiferromagnetic Pr_0.7_Ca_0.3_MnO_3 _[PCMO], is reported. The diameter of the obtained tubes varies between 6 and 8 μm, and a preferred <100> rolling direction is observed. The etching selectivity between the SRO film and the SrTiO_3 _[STO] substrate is estimated as 1:9,100. X-ray diffraction [XRD] is carried out to evaluate the original and final strain states. Unlike our previous studies using μ-focus XRD [18], diffraction is carried out for an ensemble of microtubes using a conventional single crystal diffraction beamline setup. Results clearly reveal the change in the strain state after roll-up, with the PCMO layer relaxing towards its bulk lattice parameter, whereas the upper SRO layer is compressed. Finally, μ-XRD is carried out on the same beamline, allowing for comparison of the ensemble properties with a single object. We find that a single tube can represent the ensemble indicating a good overall homogeneity of the roll-up process.

## Methods

**Figure 1:**
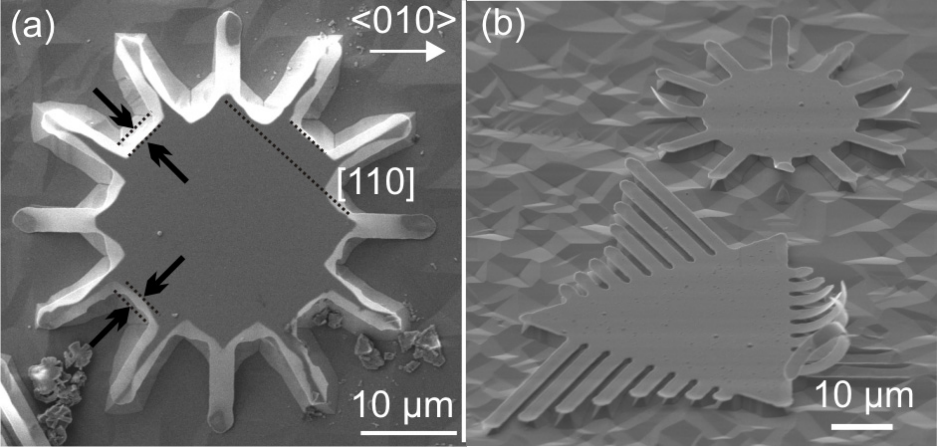
**Etching facets and curved cantilevers**. (**a**) Etching facets in <110> direction obtained by underetching a single SRO/STO(001) layer. From the etching depth, a mean etching rate of 0.55 μm/min is determined. (**b**) Curved cantilevers fabricated from trilayers. The etching time was chosen so that only those fingers in <010> directions are completely detached.

**Figure 2:**
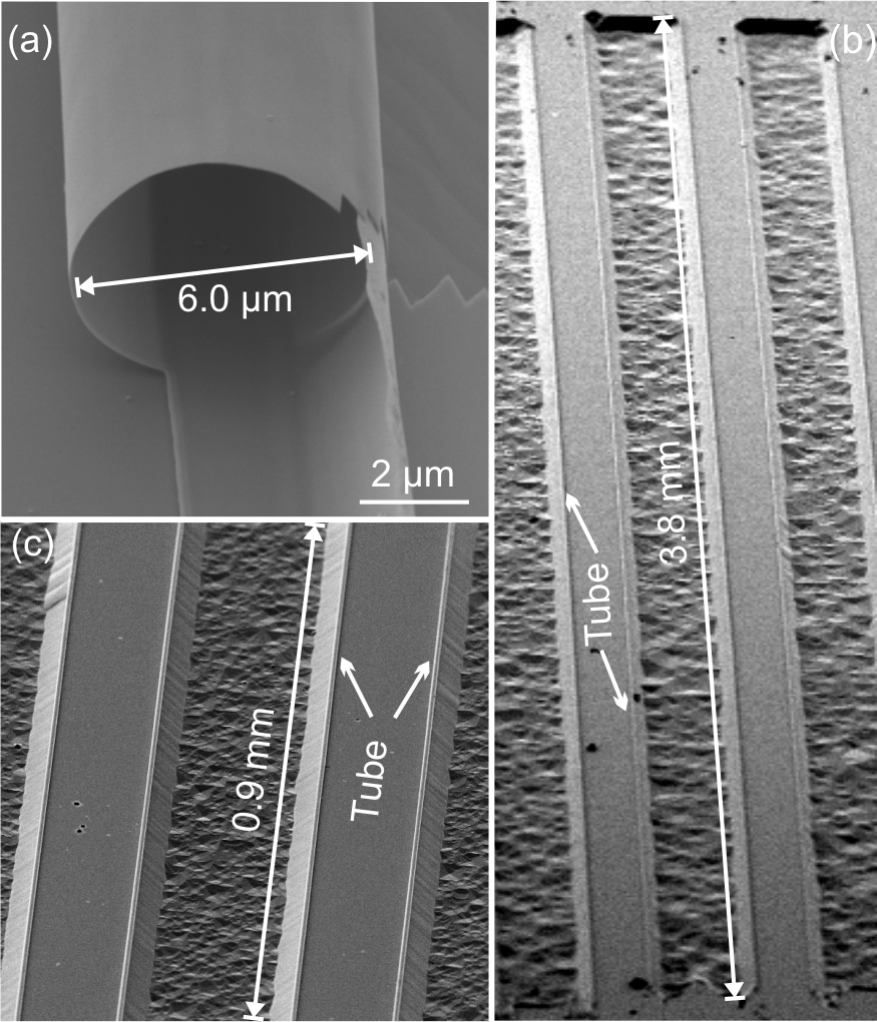
**Rolled-up SRO/PCMO/SRO microtubes**. (**a**) Rolled-up SRO/PCMO/SRO microtube with a diameter of 6.0 μm. (**b**, **c**) Positioned microtubes obtained from <100> -oriented trenches defined by optical lithography. The tubes in (b) exhibit an aspect ratio of nearly 1:700.

Several SRO/PCMO/SRO trilayers of various PCMO layer thicknesses (20 to 90 nm) were grown by off-axis pulsed laser deposition at 725°C on (001)-oriented STO substrates. A KrF excimer laser with a wavelength of 248 nm and a repetition rate of 2 Hz was used. All trilayers were grown in oxygen atmospheres of 0.14 mbar for SRO and 0.3 mbar for PCMO in order to avoid the loss of oxygen. The etching behavior was investigated for a 50-nm-thick SRO layer on a STO(001) substrate. Samples were patterned by optical lithography and ion etching. Two kinds of patterns were transferred into the layer structures: (1) a circle or triangle structure with fingers to study the etching behavior of narrow strips (Figure 1) and (2) deep-millimeter-long parallel trenches along <100>, defining the etching direction for roll-up. After patterning, the layers were released from the substrate by etching with HF (50 vol.%)/HNO_3 _(67 vol.%)/H_2_O with a ratio of 1:1:1 [20]. The obtained structures were investigated in an NVision 40 scanning electron microscope [SEM] (Carl Zeiss, Inc., Oberkochen, Germany) under different tilt angles of 0° and 54°. The height of the etched structures was measured by a Dektak 3030 profilometer (Veeco, Mannheim, Germany). Transmission electron microscopy [TEM] was carried out in a Tecnai T20 (FEI, Hillsboro, OR, USA) at 200 kV on focused-ion-beam-prepared cross sections of trilayers. Energy-dispersive X-ray [EDX] line scans were performed in a scanning TEM mode with a step width of 1 nm. XRD on an ensemble of rolled-up microtubes was carried out at the D10A-XRD2 beamline of the LNLS, Campinas (Brazil) using a 1-mm^2 ^beam, a wavelength of *λ *= 1.23985 Å, and a Pilatus 2D detector. Additionally, μ-XRD was deducted using SU-8 compound refractive lenses [21] on the D10A-XRD2 beamline with a focus of 100 × 9 μm^2^, with the larger beam dimension lying along the longitudinal tube axis. The experimental procedure was similar to the procedure for μ-XRD described before by Malachias et al. [18].

## Results and discussion

Figure 1a shows an SEM image after underetching a single SRO layer. The central part of the pattern is a circle with fingers in different crystallographic directions. The emerging etching pattern (the initial pattern is round; see Figure 1b) reveals that the solution etches anisotropically. Clear etching facets in the <110> crystal direction of the STO substrate are observed, indicating the slowest etching direction. The <100> direction is the fastest etching direction as seen in the underetched fingers (Figure 1a, b). From the etching time (2 min) and the mean underetching distance in <110> directions (1.1 μm, marked for two facets in Figure 1a), an average etching velocity of 0.55 μm/min for the <110> directions is calculated. Using the height difference between the bottom and the top of the mesa, we determine a nearly three times higher velocity of 1.45 μm/min along <001>. Since no bending or curling of the single SRO layer is observed, the strain gradient in the film is low as expected for the good lattice match between cubic lattice parameters of *a*_STO _= 3.905 Å and the pseudocubic lattice parameter *a*_SRO _= 3.928 Å [22,23].

To obtain rolled-up structures, the chemically inert SRO layer was combined with another oxide, creating a layer stack with pronounced built-in differential stress. For this purpose, trilayers with a functional oxide layer sandwiched between a bottom and a top SRO layer for protection against the acid have been grown. For the middle sandwiched layer, PCMO with a pseudocubic bulk lattice parameter of *a*_PCMO _= 3.85 Å [24] has been found to work well. Freestanding SRO/PCMO/SRO trilayer cantilever structures (with a total thickness of 120 nm) are shown in Figure 1b. The underetching was deliberately stopped after only fingers are detached in the fast etching <100> direction. The curvature of the cantilevers in Figure 1b is around 0.0625 μm^-1^. This value indicates the relatively large stiffness of the oxides.

Figure 2a shows an SEM image of the opening of a rolled-up SRO/PCMO/SRO microtube with a diameter of 6 μm. The tube has roughly performed one and a half rotation on the substrate surface. Overview images of several lithographically defined tubes of nearly 4 mm in length are shown in Figure 2b, c. The deep trenches arising from the etching time of 20 min are oriented along a <100> direction, which is assumed to be the natural rolling direction because of its maximum etching speed. The opening of the tubes is clearly observed at the beginning of the trench, indicating a good rolling behavior. Figure 2c shows a shorter tube section to better identify the tube on top of the mesa. For tubes with a diameter of 6 μm and a length of 4 mm, the aspect ratio is 1:666.

**Figure 3:**
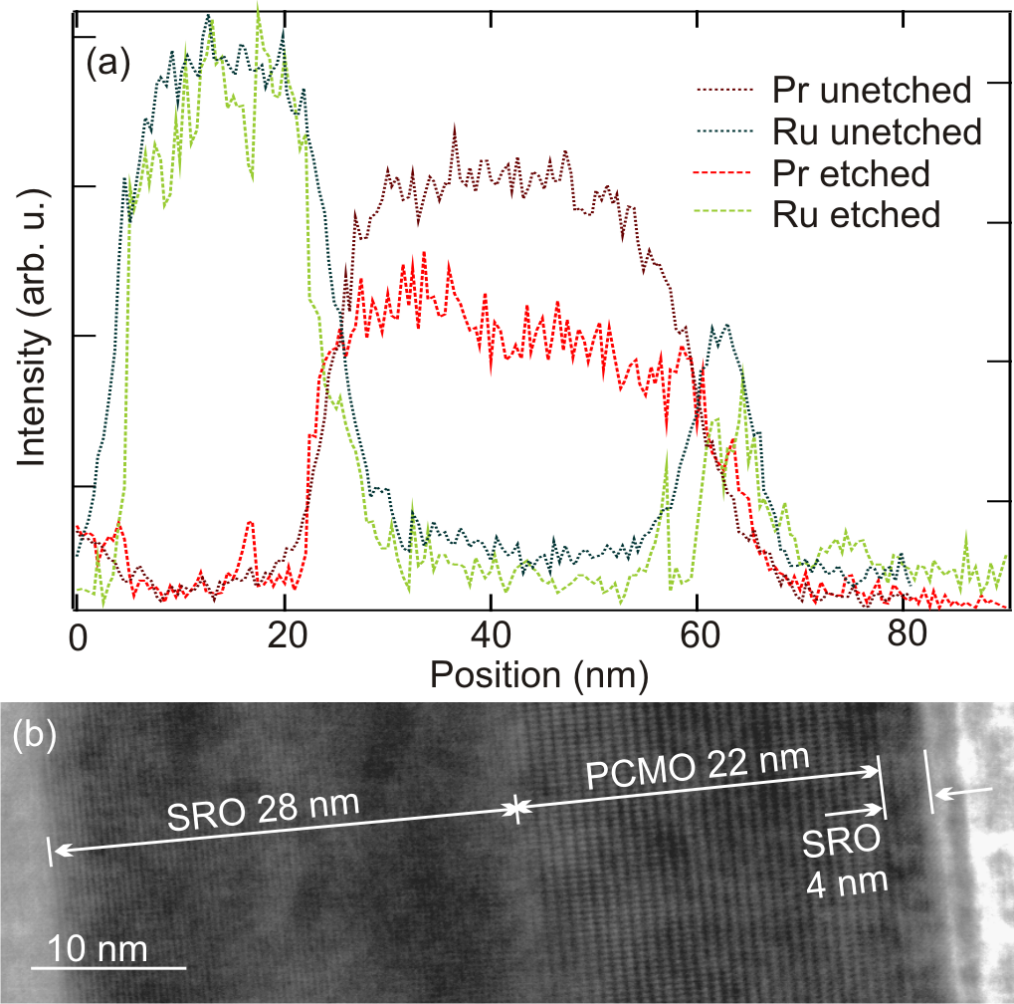
**EDX analysis and bright field TEM image**. (**a**) EDX analysis of an etched and unetched SRO/PCMO/SRO flat trilayer structure. (**b**) Bright field TEM image of the flat layer stack on the substrate after etching. The measured thicknesses were used in the simulation of the XRD spectra of the microtubes obtained from this trilayer (Figure 4).

Chemical analysis and local structural investigations have been carried out to verify that the SRO/PCMO/SRO trilayers do not suffer a chemical or structural damage during their release from the substrate. Figure 3a shows EDX line scans for Pr and Ru taken from a trilayer before and after the etching. No thickness reductions of the layers have occurred within the uncertainty (approximately 1 nm) of the measurement. The SRO top layer (4 nm) remains essentially unharmed by the etching. Using an upper limit of 1 nm for the reduction of the top layer and the applied etching time of 6 min and 10 s as well as the above determined etching rate along <100> for STO, the etching selectivity between the SRO layer and the STO substrate is above 1:9,100. Careful inspection of the trilayer cross section by high-resolution TEM indicates pseudomorphic growth of the trilayer (Figure 3b). The layer thicknesses in this sample are 28 nm SRO/22 nm PCMO/4 nm SRO, and the tube diameter is 6 μm as measured by SEM.

**Figure 4:**
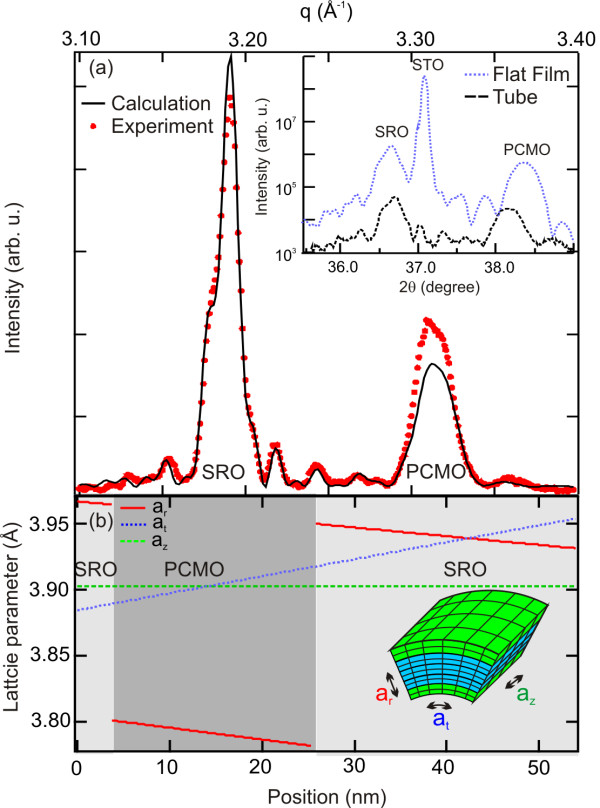
**Strain analysis of a flat SRO/PCMO/SRO layer**. (**a**) Diffraction pattern of the tube ensemble around the STO (002) reflection with experimental data (red dots) and fit (black curve, see text). The inset shows diffraction patterns of the flat film (blue, dotted line) and the rolled-up tube (black dashed line) vs. the Bragg angle around the STO (002) peak. Note the logarithmic intensity scale. (**b**) Calculated tube lattice parameters in longitudinal (*a*_z_), transversal (*a*_t_), and radial (*a*_r_) directions vs. the position measured from the inside of the tube.

In order to investigate the strain modification between a flat film and a microtube, XRD has been performed on a sample with long lithographically aligned tubes, using the geometry of Malachias et al. [18]. Figure 4a (inset) shows the diffraction patterns of the flat film and an ensemble of rolled-up microtubes in the vicinity of the STO (002) reflection. From the peak shifts, it is obvious that the PCMO undergoes a much larger strain change than the SRO. For the flat film, a pseudocubic out-of-plane lattice parameter of 3.774 Å (3.943 Å) is derived for the PCMO (SRO) layers, respectively. The SRO value agrees with that reported for pseudomorphic SRO/STO(001) films and reveals a small in-plane compression [20,22], whereas the low value for the PCMO layer results from the tensile strain induced by the SRO underlayer. For analysis, we assume a pseudomorphic trilayer according to the TEM inspection. The PCMO layer's out-of-plane (in-plane) strain is -1.97% (1.42%) using the PCMO pseudocubic bulk lattice parameter and the STO parameter as the in-plane parameter of the flat film. We use the relation *ε*_⊥ _= -2*C*_12_/*C*_11 _*ε_|| _*with the out-of-plane strain *ε*_⊥_, the in-plane strain *ε_||_*, as well as *C*_11 _and *C*_12 _as mechanical constants for the cubic lattice, giving *C*_12_*/C*_11 _~ 0.69 for PCMO. For SRO, *C*_12_*/C*_11 _= 0.513 is deduced from mechanical parameters found in the literature [25]. The diffraction pattern of the tube ensemble is calculated [18] based on the above mentioned mechanical constants, bulk lattice parameters, measured radius, and layer thicknesses. To fit the calculated curve (Figure 4a, black solid line) to the experimental data, the PCMO lattice parameter had to be changed to 3.855 Å. Considering the uncertainty of the elastic parameters and the fact that the relaxed lattice parameter of such oxide films is typically slightly larger than the bulk value, this is a realistic result. We like to point out that the layer thickness and the curvature of the rolled-up tube are similar to the ones measured in TEM and SEM. From the calculation, a longitudinal lattice parameter of *a*_z _= 3.905 Å is obtained, indicating that the tubes do not relax along their longitudinal axis. Using the calculated radial lattice parameter profile, the average radial lattice parameters *a*_r _are estimated (Figure 4b). The PCMO partially relaxes and shows *a*_r _= 3.791 Å, whereas the bottom SRO layer has nearly the same value (3.941 Å) as the flat film. The top SRO layer becomes more compressed after the roll-up, with *a*_r _= 3.966 Å. Such values strongly suggest that the strain relaxation of the PCMO is the driving force for the roll-up process.

**Figure 5:**
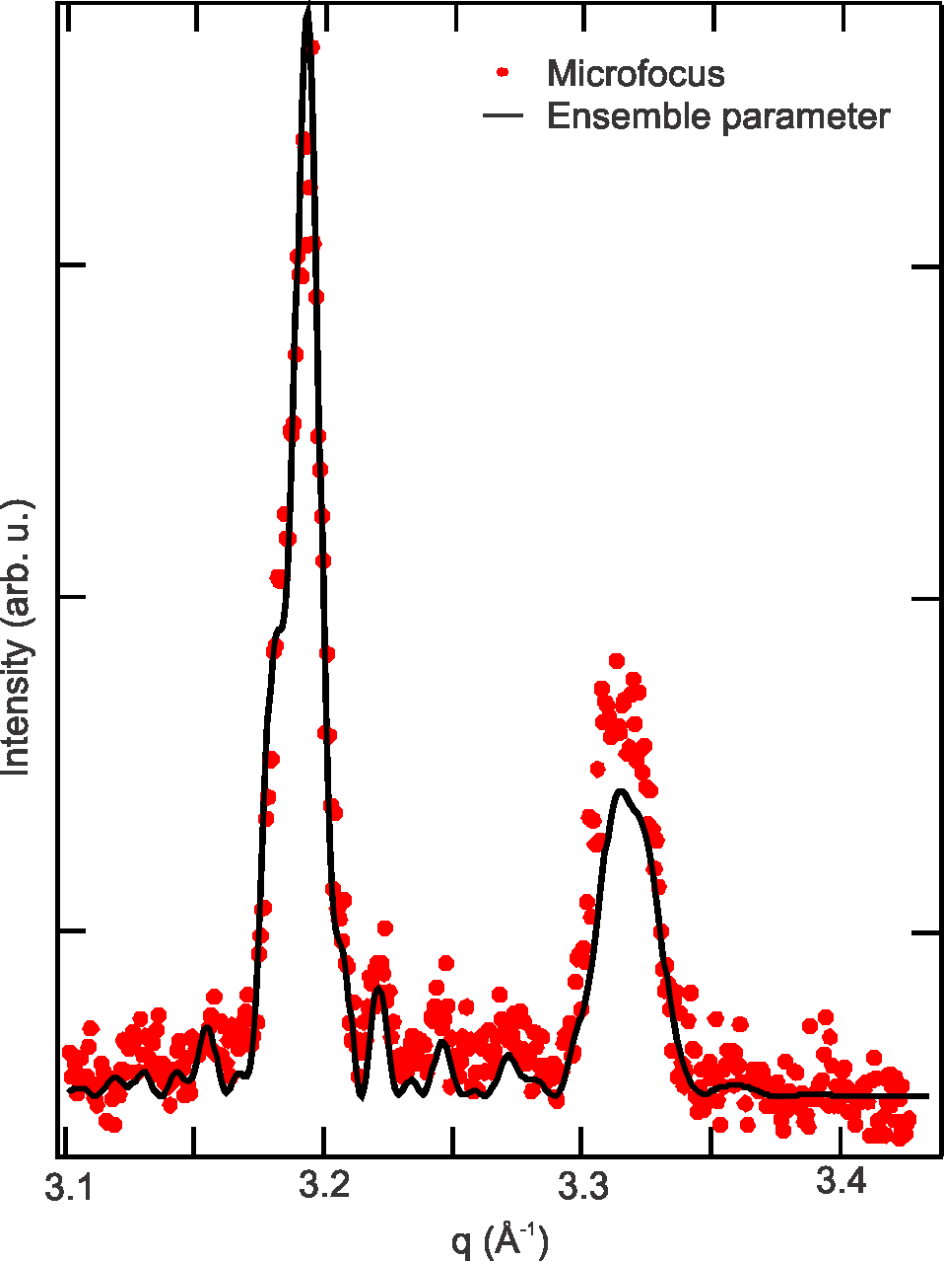
**μ-XRD pattern obtained by a 100 × 9-μm^2 ^focused beam**. The small footprint allows for probing a single tube along its axis. The experimental data (red circles) are compared to a calculated pattern (black line) using the parameters obtained from the ensemble measured in Figure 4.

In order to probe the homogeneity of the ensemble, μ-XRD was carried out with the same sample and setup. The small footprint allows for probing a single tube along its axis. Figure 5 depicts the obtained diffraction data (red circles). The diffraction pattern is compared to a calculated pattern (black line) using the parameters obtained from the ensemble shown in Figure 4. A good agreement between the calculated and experimental results is observed. We like to point out, even if the measurements exhibit some noise, most of the small features from the calculated diffraction curve are still reproduced by the experimental data. This indicates that the ensemble is well represented by a single member showing a good homogeneity of the rolled-up tubes. This conclusion is supported by the TEM investigation that provided the corrected initial layer thickness for the fitting procedure used for the ensemble data (Figure 4). As the probing volume is extremely small by TEM, the good agreement between diffraction and TEM signifies the uniformity of the rolled-up tubes.

## Conclusions

In summary, the approach of fabricating three-dimensional micro-architectures by deterministic release and rearrangement of strained films has been extended to ferroic oxides. Careful investigation of the etching behavior shows a high selectivity of 1:9,100 for an SRO film against the STO substrate. Bent-up cantilevers have been prepared by releasing pseudomorphic SRO/PCMO/SRO trilayers from an STO substrate. Patterning straight long trenches into such SRO/PCMO/SRO trilayers allows one to fabricate well-positioned rolled-up microtubes with large aspect ratios. The strain states of the oxide layers before and after roll-up have been analyzed by XRD, and the ensemble homogeneity has been checked by comparing the microdiffraction pattern of a single tube to the pattern obtained from the ensemble. This approach enables strain tailoring of three-dimensional oxide heterostructures in order to tune the magnetic, electrical, or optical properties. The layers in a microtube experience a strong linear radial strain gradient (Figure 4b) which can be tuned continuously by varying the layer thicknesses, whereas the longitudinal lattice parameter is roughly fixed to that of the substrate. The effect of such kind of strain gradient in complex ferroic oxides is rather unknown and may lead to a new behavior such as a flexoelectric effect [26]. Furthermore, cantilevers and microtubes are less clamped by the substrate. Their thus expected larger strain responses towards electric or magnetic fields may enable an improved function for strain-coupled systems such as two-phase magnetoelectric heterostructures.

## Competing interests

The authors declare that they have no competing interests.

## Authors' contributions

EW processed the samples and carried out a part of the analysis with the help of CD. PC helped with the data analysis. KB and IM grew the samples and developed the RIE etching, respectively. SB and CD carried out the SEM and prepared the TEM sample. CD did the TEM. MS, AM, and CD carried out the XRD and μ-XRD and did the analysis of the diffraction data. CD wrote the manuscript with the help of AM and KD. CD, KD, and OGS conceived and designed the experiments and supervised the work. All authors read and approved the final manuscript.
